# Importance of Macrophyte Quality in Determining Life-History Traits of the Apple Snails *Pomacea canaliculata*: Implications for Bottom-Up Management of an Invasive Herbivorous Pest in Constructed Wetlands

**DOI:** 10.3390/ijerph13030248

**Published:** 2016-02-24

**Authors:** Rita S. W. Yam, Yen-Tzu Fan, Tzu-Ting Wang

**Affiliations:** Department of Bioenvironmental Systems Engineering, National Taiwan University, Taipei 106, Taiwan; d02622007@ntu.edu.tw (Y.-T.F.); r01622027@ntu.edu.tw (T.-T.W.)

**Keywords:** apple snails, herbivory, palatability, growth, reproduction, aquatic plants, invasive species control

## Abstract

*Pomacea canaliculata* (Ampullariidae) has extensively invaded most Asian constructed wetlands and its massive herbivory of macrophytes has become a major cause of ecosystem dysfunctioning of these restored habitats. We conducted non-choice laboratory feeding experiments of *P. canaliculata* using five common macrophyte species in constructed wetlands including *Ipomoea aquatica*, *Commelina communis*, *Nymphoides coreana*, *Acorus calamus* and *Phragmites australis*. Effects of macrophytes on snail feeding, growth and fecundity responses were evaluated. Results indicated that *P. canaliculata* reared on *Ipomoea* had the highest feeding and growth rates with highest reproductive output, but all individuals fed with *Phragmites* showed lowest feeding rates and little growth with poorest reproductive output. Plant N and P contents were important for enhancing palatability, supporting growth and offspring quantity of *P. canaliculata*, whilst toughness, cellulose and phenolics had critically deterrent effects on various life-history traits. Although snail offspring quality was generally consistent regardless of maternal feeding conditions, the reduced growth and offspring quantity of the poorly-fed snails in constructed wetlands dominated by the less-palatable macrophytes could limit the invasive success of *P. canaliculata*. Effective bottom-up control of *P. canaliculata* in constructed wetlands should involve selective planting strategy using macrophytes with low nutrient and high toughness, cellulose and phenolic contents.

## 1. Introduction 

Constructed wetlands are engineered systems extensively established worldwide in recent decades as the mitigation and restoration measures for the lost and degraded natural wetlands due to human activities. They are designed and built to mimic multiple natural processes serving for various purposes including wastewater treatment, support of biodiversity and biomass production, flood retention, aesthetic enhancement for landscape, space provision for public recreation and environmental education [1,2,3,4,5,6]. However, constructed wetlands are highly susceptible to invasion and dominance of competitive-superior invasive species due to their relatively low ecosystem structural complexity related to short establishment time and intensive human disturbances [7]. This has caused serious threats to the restoration success of constructed wetlands by hampering many important ecosystem functions provided by the local biological assemblages, e.g., [8,9,10,11]. 

The apple snail *Pomacea canaliculata* (Gastropoda: Ampullariidae), which is native from South and Central America, has been identified as one of the 100 worst invaders worldwide and caused numerous impacts on the invaded wetlands in particular the Asian region for >40 years [8,12,13,14,15]. The fast growth, iteroparous reproduction, high fecundity and lack of effective predators and competitors of this gonochoristic gastropod have clearly contributed to its invasion success and strong competitiveness in most Asian wetlands [15,16,17,18]. The abilities of amphibious respiration and tolerance to water pollution further facilitate its superior adaptation in the eutrophic and hypoxic environment of constructed wetlands. Recent studies have confirmed the strong capability of the macrophytophagous *P. canaliculata* for reducing macrophyte biomass [17,19,20]. In fact, macrophytes represent one of the indispensable components of constructed wetlands due to not only their important properties associated with wastewater treatment processes (such as physical filtration, erosion control, provision of surface area for microbial colonisation, nutrient uptake and oxygen release), but also their functions as habitat provision for wildlife, support of primary productivity, regulation of nutrient cycling, and aesthetic enhancement [1,4,5,6,21]. Therefore, the macrophyte herbivory of *P. canaliculata* could result in serious impacts on many ecosystem functions such as lowering water purification effectiveness, altering nutrient cycling and decreasing availability of habitat and food sources to other species in constructed wetlands. 

Conventional physical and chemical techniques have been widely used to eliminate *P. canaliculata* from the invaded wetlands, e.g., [22,23,24]. But, these methods did not provide any sustainable solution to control this snail and exhibited little control effectiveness despite the high operation cost involved. Recently, biological control has been considered as the more preferable management strategy for *P. canaliculata* in wetland ecosystems. However, this has only been limited to the top-down approach by introducing snail predators, such as common carp (*Cyprinus carpio*), and mallard duck (*Anas platyrhynchos*), to regulate the field populations of *P. canaliculata* [25,26,27]. Due to the non-selective feeding habit of these snail predators, the non-target flora and fauna could be inevitably damaged resulting in further negative impacts to ecosystems functions. 

On the contrary, the bottom-up approach of biological pest control involves alterations of the abundance of the targeted species by changing the abundance of food and habitat availability [28,29]. So far, bottom-up control on the apple snails in wetland ecosystems has received little attention [30] despite that an early study unintentionally discovered that regular habitat disturbance by crop rotation was an effective measure of reducing the density of *P. canaliculata* in paddy fields from Japan [31]. Recent laboratory investigations on feeding preference of the apple snails have revealed that the macrophyte palatability was highly linked to plant chemistry and physical structure [16,30,32,33,34]. These findings provided important details on the general influence of macrophyte characteristics on the snail herbivory and life-history traits. However, the characteristics of macrophytes grown in constructed wetlands could be strongly affected by the elevated levels of nutrients and pollutants from the wastewater input [35,36]; their influence on the feeding and life-history traits of *P. canaliculata* could be different from those plants inhabiting in natural wetlands or paddy fields [30,32,33]. Therefore, it would be essential to understand which macrophyte species or what plant characteristics contribute to the low palatability and poor support to the feeding and life-history traits of *P. canaliculata* in constructed wetlands. Although the current selection basis for macrophyte species planted in constructed wetlands is primarily based on their capability of nutrient retention and tolerance to adverse conditions in local environment, it would be important to incorporate the bottom-up control of the apple snails into the macrophyte planting design for constructed wetlands. Yet, the scientific knowledge necessary for devising the bottom-up control techniques of *P. canaliculata* has remained unclear. 

Constructed wetlands have been widely established in Taiwan since the late 1990s. About 100 major constructed wetlands are built on river floodplains. Due to the rapid economic development and urbanization, these constructed wetlands serve the primary functions as mitigation sites for the lost and degraded natural wetlands, economical systems for municipal wastewater treatment and habitat provision for wildlife. Moreover, as influenced by the subtropical climate, heavy rainfall associated with monsoons and typhoons commonly occurs during summer (April to September) in Taiwan. Constructed wetlands also play important roles on flood abatement. However, the impact of intensive herbivory from the dominant pest *P. canaliculata* has seriously damaged the restored macrophytes, hampered the associated ecosystem functions and restoration success of the constructed wetlands [11,37]. Thus, there is a pressing need to devise appropriate control measures of *P. canaliculata* in the constructed wetlands. In this study, we quantified the palatability to *P. canaliculata* of five common macrophyte species from constructed wetlands in Taiwan based on their feeding rates in the non-choice laboratory feeding experiment. Also, the snail growth and fecundity performance were compared among the five macrophyte feeding treatments. Finally, the physical and chemical characteristics of the five study macrophyte species were analysed to evaluate their influence on the life-history traits of *P. canaliculata*. Our results would provide important knowledge for the selective criteria of macrophyte species suitable for planting in constructed wetlands to control the invasive *P. canaliculata* and reduce its herbivory impact by the bottom-up management approach.

## 2. Materials and Methods

### 2.1. Field Collection of Snails and Macrophyte Leaves

Sampling of experimental individuals of *Pomacea canaliculata* was undertaken in Daniaopin Free Water Surface Constructed Wetland, New Taipei City, N. Taiwan (121°26’–42’E, 24°59’–98’N; 38 ha), during August–September 2012. Climate in N. Taiwan is subtropical with hot summer from April to September and cold winter from October to March. The apple snails are commonly seen in constructed wetlands during summer. We collected 500 individuals of *P. canaliculata* in the present study including 100 individuals with random shell heights (SH; vertical distance from the apex to the aperture) for establishing the regression relationship between snail biomass and different morphological parameters, 300 individuals with SH between 20–25 mm for the growth experiment, and 100 individuals with SH > 25 mm for the fecundity experiment. As *P. canaliculata* is an invasive pest, field collection was not restricted by any legislation. The animal protocols used in this work were approved by the Institutional Animal Care and Use Committee (IACUC) of National Taiwan University (Approval no. 98–40). They are in accordance with the guidelines of Aquatic Invertebrate Handling by IACUC and Article 15–17 of Chapter 3 Scientific Application of Animals from the Animal Protection Law (Law no. 10400014321). 

In this study, five macrophyte species in constructed wetlands, including *Ipomoea aquatica* Forsk. (Convolvulaceae), *Commelina communis* L. (Commelinaceae), *Nymphoides coreana* (H. Lev.) (Menyanthaceae), *Acorus calamus* L. (Acoraceae) and *Phragmites australis* (Cav.) Trin ex Steud. (Poaceae), were used as five feeding treatments for the snail growth and fecundity experiments (Table 1). All five study macrophytes showed consistent dominance in most constructed wetlands in Taiwan. *Ipomoea*, *Nymphoides*, *Acorus* and *Phragmites* were commonly planted species, but *Commelina* was a widespread weed in our sampling site and other constructed wetlands. Only newly-grown leaves from adults plants (not seedlings) of all five macrophyte species were used for the snail growth and fecundity experiments to avoid the confounding effect of snail feeding preferences due to plant ages. Also, fresh macrophyte leaves were collected from the sampling site every 2 days during the experimental period to avoid any confounding effect on feeding preference because of variation in plant freshness. In the laboratory, all leaves were gently washed with deionised water and blotted dry, their standard weight was measured to 50 ± 0.1 g, and they were then stored at 7 °C for use in the snail growth and fecundity experiments. Leaves used for chemical analysis were dried at 50 °C to constant weight (±0.01 g), homogenized (particle size ≈ 0.5 mm) and stored in a desiccator prior to the analysis.

### 2.2. Regression between Snail Biomass and Different Morphological Parameters 

In addition to SH, dry mass (DM) of snails were used to represent snail biomass as the growth indicators in this study. As the shells of freshwater snails consist of >95% inorganic carbon, *i.e*., calcium carbonate [38], shells were excluded for the measurement of DM of snails. Since these measurements involved killing the animals, obtaining snail DM during the growth experiment was not possible. In the laboratory, three morphological parameters including SH (±0.01 mm), wet weight (WW; ±0.01 g) and aperture width (AW; ±0.01 mm) were measured for the 100 snails with random SH (1.0–60.0 mm) collected from the sampling site. The snails were killed by refrigeration. Each snail was dissected, and the muscular body mass was separated from the shell. The WW was measured for both the shell and soft body. DM was recorded after oven-drying at 60 °C to constant weight (~72 h). Simple linear regressions were established between snail biomass and the three morphological parameters, including DM–AW, DM–SH, DM–WW, prior to the snail growth experiment. Results showed that log SH could explain the highest variability in the log DM of snails (log DM = 3.1631 log SH = 1.7418; r^2^ = 0.95; *p* < 0.001). Therefore, SH of the apple snails were measured during the growth experiment and used as the non-destructive morphological indicator for DM conversion.

### 2.3. Experiment 1: Snail Feeding and Growth Responses

Snail feeding and growth responses towards the five study macrophytes was tested by presenting leaves of each macrophyte to snails in the non-choice experiments. Prior to the experiment, all experimental snails were first acclimated with no food in laboratory aquaria containing aerated tap water at 25 ± 1 °C for 24 h, and individuals with obvious injuries were excluded. Six replicates of experimental chambers (size = 30 × 20 × 20 cm; water depth = 5 cm) were used for each macrophyte feeding treatment, and each chamber contained nine snails (20–25 mm SH) and 50 g of fresh leaves. Also, three replicates of control for each study macrophyte were set up with no snails. The snails were reared at 25 ± 1 °C and a photoperiod of 12-h light:12-h dark. Every two days, fresh macrophyte leaves and water were renewed, and any faeces of snails were removed in each experimental chamber. The remaining leaves in each experimental chamber were oven-dried at 50 °C to constant weight and their DM were measured (±0.01 g). Snail feeding rate (g·g^−1^·d^−1^) was calculated with respect to the snail DM (determined from the SH-DM regression above) *per* day. The SH of each snail was measured every 6 days. At the end of the 60-day experimental period, the final SH and DM of each snail were determined. The snail growth rate from each experimental chamber was calculated in terms of SH (SH-growth; mm·d^−1^) and DM (DM-growth; mg·d^−1^).

### 2.4. Experiment 2: Snail Fecundity Performance 

The snail fecundity performance upon feeding with each of the five study macrophytes was investigated by placing one adult male and one adult female snail (SH > 2.5 cm) in the experimental chamber. Sex determination of adult *P. canaliculata* followed the method in [39]. Each macrophyte species was tested in six replicates of experimental chambers. All conditions were maintained identical to those of Experiment 1. All experimental chambers were checked every day, any egg clutch laid was removed immediately. However, any egg laid by snails during the first six days of the experiment was excluded to avoid the confounding effect on snail fecundity performance due to the fertilized eggs produced before the experiment. Then, each egg clutch produced by snails was carefully transferred onto the surface of a plastic board tilted in a separate tank with 5-cm water depth and maintained at 25 ± 1 °C. The snail fecundity performance upon different feeding treatments was determined based on the female reproductive output (*i.e*., quantity of potential offspring) that was evaluated by the number of clutches and clutch size (number of eggs *per* clutch) produced per female snails; and the quality of offspring that was assessed by the egg hatching success (%) and biomass of individual egg (DM-egg; mg) and neonate (DM-neonate; mg) to the nearest 0.1 mg. This experiment was conducted for 60 days, but snail fecundity performance upon different feeding treatments was assessed for a 54-day period (without the consideration of the first six days). After the experiment, all parent snails were dissected for confirmation of their sexes.

### 2.5. Physical and Chemical Characteristics of Macrophytes

In this study, six physical and chemical characteristics were analysed for the leaves of *Ipomoea*, *Commelina*, *Nymphoides*, *Acorus* and *Phragmites* to evaluate their influence on feeding rate, growth and fecundity responses of *P. canaliculata*. The nutrient levels of leaves were indicated by the contents of total nitrogen (N) and total phosphorus (P). The levels of defensive chemicals of leaves were assessed based on the content of total phenolics. The physical resistance of leaves to herbivory was examined according to the contents of lignin and cellulose, and the physical toughness. For each study macrophyte species, three replicate samples were analysed for individual physical and chemical characteristics. The combined method for N and P determination was used to analyse each leaf sample following the Kjeldahl-N method [40]. A 5 mg (±0.1 mg) homogenized leaf sample was placed in 25 mL of distilled water followed by the addition of 1 mL 98% H_2_SO_4_ with 0.2 mL CuSO_4_ (10%) in an Erlenmeyer flask. The sample was pre-digested at 100 °C for 1 h and then at 375 °C for 2 h using the micro-Kjeldahl apparatus. After cooling, NaOH was added to the digestate until the pH fell between 5–6, and pH could not exceed 6 to avoid any precipitation of dissolved phosphorus. The digestate was filtered through the GF/C filter (Whatman^®^ Glass microfibre filter, Grade GF/C) and the volume was made up to 100 mL. The N and P concentrations were determined by the indophenol- and ascorbic-acid-methods respectively [41]. 

For measurement of the total phenolic content, the homogenized leaf sample (200 mg) was extracted with 10 mL 70% acetone (v/v) for 1 h at 4 °C and centrifuged at 15,000 g for 10–20 min. The total phenolic content was measured with Folin-Ciocalteu's reagent, with ferulic acid as standard, according to [42]. An 0.5 mL portion of the supernatant from the centrifuged solution was made up to 1.0 mL with distilled water, and reacted with 5 mL Na_2_CO_3_ (2%) in NaOH (0.1 N) and 0.5 mL of Folin-Ciocalteu’s reagent. The solution was allowed for 120-min incubation, and absorbance at 720 nm was measured by a spectrophotometer. 

The Cellulose content of each leaf sample was evaluated according to [43] with modifications. A 250-mg of homogenized leaf sample was suspended in 20 mL of acid detergent solution (0.5 M H_2_SO_4_ and 20 g·L^−1^ CTAB) with 0.4 ml Decahydronaphtalene, and digested at 100 °C for 1 h. After cooling, the solution was centrifuged at 12,000 g for 10 min. The pellet material, *i.e*., acid-detergent-fibre (ADF), was collected, washed in 30 mL hot distilled water (90–100 °C) and re-centrifuged until the pH of supernatant >6. Then, the pellet was washed with acetone, centrifuged, oven-dried at 105 °C for 24 h and weighed after cooling in a desiccator for 1 h. The ADF was comprised of lignin, cellulose and some ashes. The ADF was hydrolysed by adding 72% H_2_SO_4_ in small portions several times at 20–23 °C water bath with constant stirring for 3 h. The solution was centrifuged at 12,000 g for 10 min. The pellet was collected, washed in 30 mL hot distilled water until free from acid, oven-dried at 105 °C for 24 h and weighed after cooling in a desiccator for 1 h. This dried sample was then ashed at 550 °C for 3 h. The cellulose content was determined by weight loss due to acid-detergent treatment whereas the lignin content was evaluated by weight loss due to ashing after acid-detergent treatment. All chemical contents, including N, P, phenolics, lignin and cellulose, of study leaf species were expressed as percentage with respect to the dry weight of leaf sample.

The physical toughness of fresh leaves of the five study macrophyte species were determined using a tearing device described in [44]. The leaf toughness was measured as the resistance to tearing. The fresh leaf sample was cut into disc (~10-mm diameter) which was secured between two pegs with one fixed to a ring stand by a clamp and another connected to a cup via a string passing through a pulley. Sand was gradually added to the cup until the accumulated weight tore apart the leaf disc. The combined weight of cup and sand was recorded as the leaf toughness (±0.01 g). 

### 2.6. Statistical Analysis

The snail consumption and SH-growth on each of the five study macrophyte species during the study period were compared using one-way ANOVA. The feeding rates, growth rates (SH-growth and DM-growth), and fecundity performance (clutch size, number of clutches produced per female, DM-egg, egg hatching success and DM-neonate) of *P. canaliculata* were compared among the feeding treatments of five macrophyte species using one-way ANOVA followed by *post hoc* Tukey tests. The difference in physical and chemical characteristics among the five study macrophytes were also determined by one-way ANOVA and Tukey tests. Pearson correlation was conducted to evaluate the association between macrophyte characteristics. As variations in the feeding, growth and fecundity responses of *P. canaliculata* were generally poorly explained by single macrophyte characteristic, best subset regression was performed to search for the subset of the most influential plant characteristics explaining the highest variability (with the lowest Mallows’ *C_p_* statistic) of the snail feeding, growth and fecundity responses. Statistical analyses were carried out using Minitab 16.0 and SPSS 16 packages. 

## 3. Results

### 3.1. Snail Feeding and Growth Responses

**Figure 1:**
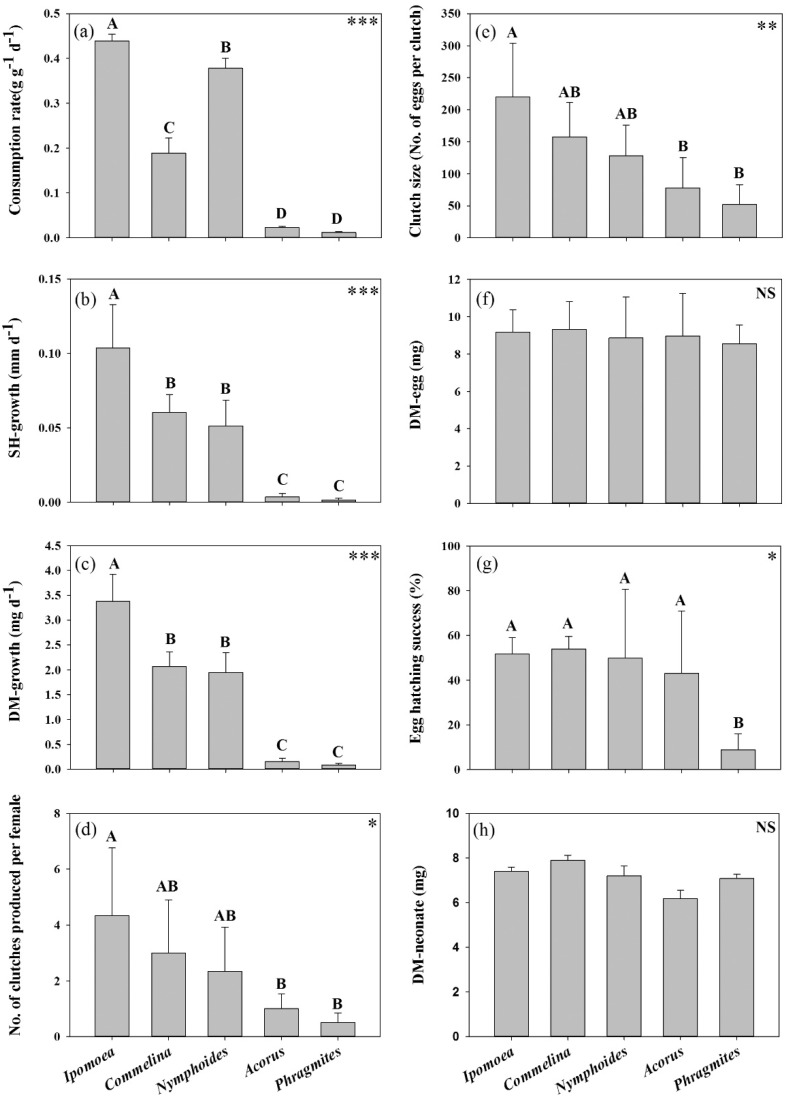
Mean (+ SD) of different responses of *Pomacea canaliculata* upon fed with the five study macrophyte species: (**a**) feeding rate; (**b**) SH-growth; (**c**) DM-growth; (**d**) Number of clutches produced per female; (**e**) Clutch size; (**f**) DM-egg; (**g**) Egg hatching success; (**h**) DM-neonate. * *p* < 0.05; ** *p* < 0.01; *** *p* < 0.001, ^NS^ no significance.

*Pomacea canaliculata* demonstrated significant consumption of all study macrophyte species (*p* < 0.001; one-way ANOVA; Table 2). Snails showed the highest mean percentage consumption of *Ipomoea* (57.18% ± 27.25%) whereas the lowest consumption of *Acorus* (2.66% ± 1.07%) and *Phragmites* (1.33% ± 0.79%) during the experiment. Their feeding rates also varied significantly among the five feeding treatments (*F* = 1560.84, *p* < 0.001; one-way ANOVA; Figure 1a). Highest palatability to snails was exhibited by *Ipomoea* but lowest by *Acorus* and *Phragmites* as *Ipomoea* supported the highest snail feeding rates (0.439 ± 0.015 g·g^−1^·d^−1^) and it was approximately 37× and 19× that for *Phragmites* and *Acorus* respectively (Tukey test: *Ipomoea* > *Nymphoides > Commelina* > *Acorus* = *Phragmites*; *p* < 0.001). 

Results of one-way ANOVA showed significant difference in SH-growth of *P. canaliculata* among the five macrophyte treatments (*F* = 37.14, *p* < 0.001; Figure 1b). In fact, only the feeding treatments of *Ipomoea*, *Commelina* and *Nymphoides* supported significant SH-growth (*p* < 0.001; one-way ANOVA; Table 2). In contrast, SH increment was not detected for the snails reared on *Acorus* and *Phragmites* throughout the experimental period. Highest SH-growth was exhibited by the snails fed with *Ipomoea* (0.104 ± 0.029 mm·d^−1^) and it was >10× higher than the individuals reared on *Acorus* and *Phragmites* (*Ipomoea* > *Commelina* = *Nymphoides* > *Acorus* = *Phragmites*; Tukey test; *p* < 0.001; Figure 1b). In this study, a similar trend was seen between snail SH-growth and DM-growth among the five feeding treatments (*F* = 17.03, *p* < 0.001; one-way ANOVA; Figure 1c). The DM-growth of *P. canaliculata* was highest for *Ipomoea* (3.377 ± 0.540 mg·d^−1^), and it was ~40% higher than that of snails fed with *Commelina* and *Nymphoides,* and double that of individuals reared on *Acorus* and *Phragmites*.

### 3.2. Snail Fecundity Performance

Both the number of clutches produced per female (*F* = 4.15, *p* < 0.05; one-way ANOVA; Figure 1d) and the clutch size (*F* = 5.94, *p* < 0.01; one-way ANOVA; Figure 1e) showed consistent variation among the five feeding treatments (*Ipomoea* ≥ *Commelina* = *Nymphoides* ≥ *Acorus* = *Phragmites*; Tukey test; *p* < 0.05) during the 54-day period for actual assessment of snail fecundity performance (noted that any egg laid by snails in the first six days before this 54-day period was excluded). The females fed with *Ipomoea* produced two to seven egg clutches during the 54-day experimental period such that one clutch was generally produced per one to two weeks. The snails reared on *Commelina* and *Nymphoides* produced one to five clutches during the experimental period, and thus one clutch was laid per two to three weeks. In contrast, the experimental individuals fed with *Phragmites* and *Acorus* only produced an average of 0.5–1 egg clutch during the first two weeks of the experiment. As similar to the growth response, *Ipomoea* supported the highest reproduction output of female *P. canaliculata*. The mean number of clutches produced by the females fed with *Ipomoea* was 4.33 ± 2.42, and their mean clutch size was 220.14 ± 83.64 eggs. The snails fed with *Commelina* and *Nymphoides* exhibited similar clutch characteristics such that their number of clutch per female snail and clutch size were 30%–50% less than the individuals reared on *Ipomoea*. Females reared on *Phragmites* and *Acorus* produced the lowest mean number of clutches and clutch size which were 70%–90% less than that produced from *Ipomoea* treatment. Also, about half of the female snails from the *Phragmites* and *Acorus* treatments did not produce any egg clutch during the study period.

The three attributes for offspring quality were highly consistent regardless the maternal feeding conditions in different macrophyte treatments except that significant lower hatching success of the eggs produced by the female snails fed with *Phragmites* (8.85% ± 7.11%) as compared to the other four macrophyte treatments (*F* = 5.47, *p* < 0.01; one-way ANOVA; Figure 1g). The females reared on *Ipomoea*, *Commelina*, *Nymphoides* and *Acorus* produced eggs with similar hatching success which were varied between 43.02%–53.9% (*Ipomoea* = *Commelina* = *Nymphoides* = *Acorus* > *Phragmites*; Tukey test; *p* < 0.01). Moreover, no significant difference was revealed in both DM-egg (range = 8.55–9.32 mg) and DM-neonate (range = 6.18–7.90 mg) produced by females among the five feeding treatments (*F* = 0.26, *p* > 0.05; one-way ANOVA; Figure 1f,h).

### 3.3. Physical and Chemical Characteristics of Macrophytes

The five study macrophyte species revealed significant variation in their physical and chemical characteristics (Table 1). *Ipomoea*, *Commelina* and *Nymphoides* were generally higher in nutrient contents (N and P) but lower in physical (toughness, cellulose, lignin) and chemical defensive characteristics (phenolics), whilst *Acorus* and *Phragmites* showed the opposite patterns. *Ipomoea* and *Commelina* exhibited the highest N and P content ranging from 3.11%–5.01% and 0.48%–0.86% respectively, whereas *Acorus* and *Phragmites* showed the lowest nutrient contents. Highest contents of phenolics were measured for *Acorus* and *Nymphoides* (2.51%–3.17%), but lowest level was determined for *Phragmites* (0.29%–0.31%). *Phragmites* had the highest toughness with highest cellulose and lignin contents (toughness = 192.14–271.88 g; cellulose = 20.17%–24.53%; lignin = 6.15%–7.36%) among all study plants. In addition, our results showed significantly positive correlation between N and P of the macrophyte leaves (*r* = 0.831; Table 3). The nutrient contents were negatively correlated to most defensive characteristics. N was negatively correlated to all defensive characteristics (*r* = −0.39 to −0.72) whereas P was only negatively correlated to the physical defensive characteristics (*r* = −0.42 to −0.71). Both plant cellulose and lignin contents were positively correlated with toughness (*r* ≥ 0.55) but there was no significant correlation between cellulose and lignin. 

### 3.4. Influence of Macrophyte Characteristics on Snail Life-History Traits 

According to the best subset regression model, snail feeding rate was best predicted by positive association with P content and negative association with phenolics and cellulose (Adj. *R*^2^ = 0.78; Table 4). The models of the two snail growth attributes were similar, both SH-growth and DM-growth were highly explained by positive association with P and negative association with cellulose and toughness. However, influences of the macrophyte characteristics on the five attributes for fecundity performance were less consistent. Both the number of clutches per female snail and clutch size were best explained by positive association with N and negative association with phenolics and cellulose. The egg hatching success was significantly predicted by negative association with phenolic and cellulose contents. However, snail DM-egg and DM-neonate could not be explained by any measured macrophyte characteristics in this study as none of these variables were selected as predictor in the model. Results indicated that the adjusted *R*^2^ values were higher for the snail feeding and growth responses (adjusted *R*^2^ = 0.76–0.84), but lower for the fecundity attributes (adjusted *R*^2^ = 0.30–0.60). 

## 4. Discussion 

### 4.1. Snail Feeding and Growth Performance

The present non-choice feeding experiments revealed that *Pomacea canaliculata* exhibited different feeding rates on the five common macrophytes in constructed wetlands and their life-history traits varied greatly upon feeding with different plants. The apple snails reared on *Ipomoea* had the highest feeding and growth rates with highest reproductive output, but all individuals fed with *Acorus* and *Phragmites* showed lowest feeding rates and little growth with poorest offspring quantity. It was difficult to determine if the variation in snail growth and fecundity responses resulted from different feeding rates or plant characteristics. However, when the snail responses were compared between the feeding treatments of *Nymphoides* and *Commelina*, it was obvious that their growth and clutch production patterns were highly similar though snail feeding rate on *Commelina* was only half of that on *Nymphoides*. This suggested that feeding rates of *P. canaliculata* did not necessarily associate with growth rates and fecundity performance. Instead, macrophyte characteristics would be more important determinants for the snail growth and fecundity. Previous findings indicated that plant protein contents could play important roles on the fitness of invertebrate herbivores including snails, insects and crustaceans (*i.e*., survival, growth and reproduction) and thus they had strong N- and P-dependence from plants for protein synthesis [45,46]. Several studies on *Pomacea* spp. confirmed that macrophyte N and P contents were consistently important for supporting their feeding and growth [16,20,30,32]. However, only macrophyte P content was selected out of the two nutrient characteristics as the key determinant for feeding and growth rates of *P. canaliculata* in our study. Such insignificant association with N content was, however, relevant to other studies on macrophyte herbivory by invertebrates of which feeding could be inhibited by the presence of some levels of deterrents regardless of plant nutrient content [33,46,47]. Moreover, all five macrophytes in this study had relatively high N content (2.18%–4.28%) as compared to the published data from other wild macrophytes, e.g., [30,32,46]. Since N was needed as an essential element by snails for metabolic requirements [34,45], the macrophyte food sources appeared to provide sufficient N to support the survival of *P. canaliculata*. On the contrary, P was an essential but limiting resource for protein synthesis in plants and animals of most aquatic ecosystems, the ambient P concentration in our sampling site was generally low (0.05–0.58 mg/L) [48]. Therefore, macrophytes with high P content could enhance the plant palatability and growth of *P. canaliculata* in the environment where N was not limited, e.g., constructed wetlands. 

In fact, accumulated studies on macrophyte effects of most invertebrate herbivores emphasized that plant nutrient contents were only secondarily important for determining plant palatability, however the levels of chemical and physical defensive characteristics were of primary importance for determining their feeding preferences and growth rates [47,49,50]. It has been reported that the food selection, feeding and growth of herbivorous snails, including *P. canaliculata*, *P. paludosa, Lymnaea stagnalis* (Lymnaeidae) and *Littoraria irrorata* (Littorinidae), were affected by more than one plant characteristics [32,33,51,52,53]. Thus, both nutrient content and defensive characteristics of macrophytes were considered strongly influential on the feeding and growth rates of *P. canaliculata* in our study. As generalist herbivores, *P. canaliculata* tended to feed on plants with high nutrients (benefits) and low defensive characteristics (cost) to maximise their energy intake and fitness according to the optimal foraging theory, see [45,46,54]. An earlier study on rice system also demonstrated that *P. canaliculata* had significantly higher feeding performance on young plants [22]. Such selective herbivory indicated that physical defensive characteristics, in addition to nutrient contents, of macrophytes could play an important role on feeding and growth of *P. canaliculata.* Tissue toughness or dry-matter content which was largely associated with cell-wall components, *i.e*., cellulose content, has been commonly regarded as a strong determinant for macrophyte palatability to snails and other invertebrates [52,55,56]. *P. canaliculata* exhibited low palatability of *Phragmites* leaves, but more than five-fold increase in the snail consumption of the reconstituted-*Phragmites*, which was made by mixing homogenized dried *Phragmites* leaves with agar containing the same chemical contents as natural *Phragmites* but damaged physical structure [30]. Hence, this confirmed the critically deterrent effect of physical defensive characteristics of macrophytes, particularly for *Phragmites* that contained only low level of phenolics, on the feeding and growth performance of *P. canaliculata* in the present study. 

Plant phenolic content was generally considered to have inhibition effects on the feeding and growth of herbivorous snails, but there was no consistent findings of its deterrent effect on the life-history traits of *Pomacea* spp. Both *P. maculata* and *P. canaliculata* exhibited low sensitivity to defensive chemicals, [57,58]. However, feeding preference of *Pomacea* spp. generally responded primarily to plant chemistry [33]. Several studies also suggested that high plant phenolics of macrophytes could be the key defensive characteristic against herbivory of *P. canaliculata*, the consumption, survival and growth were strongly determined by phenolics [30,32,34]. Our results showed that phenolics was one of the best explanatory variables for snail feeding rate despite that the phenolic contents of all study macrophytes were relatively low (<3.1%) as compared to other macrophytes from wild populations. In fact, other non-phenolic defensive chemicals might be present in the macrophyte tissues deterring the snail feeding and growth [55], and further study would be needed for better understanding of macrophyte effects on snail herbivory by analysis of other secondary metabolites and their defensive characteristics.

Apple snails are capable of undergoing aestivation to enter a state of dormancy with lowered metabolic rate during unfavourable conditions such as food scarcity, low temperature and drought as their adaptation strategy in the highly variable wetland environment [13,14,16,34,59]. The aestivated snails could persist through periods of low food availability as the result of increasing their food assimilation by reducing their feeding rates and increasing food retention time in their digestive systems. In our study, *P. canaliculata* fed with nutrient-poor and highly-defensive *Phragmites* and *Acorus* showed little feeding and growth, and lowest reproductive output. Though no mortality of the aestivated snails was observed throughout our experiments, all experimental individuals reared on these two plants became less active. This was because of under-nutrition and accumulation of uric acid in the snail bodies, and thus their mortality risk could be increased for long periods of aestivation [14,59,60,61]. Previous studies found that *P. canaliculata* underwent starvation and survived for 2–20 weeks during food deprivation, e.g., [16,32]. *Pomacea canaliculata* also exhibited aestivation behavior with low growth and high mortality in a 30-day feeding treatment with a low N-content plant (~0.5%) [34]. Therefore, macrophyte species exhibiting critical minimum level of food quality, *i.e*., elevated levels of defensive characteristics with poor nutrition, could strongly inhibit the feeding and growth of *P. canaliculata* given that no other macrophyte species or food types are available in the environment. However, further studies investigating the effect of feeding preference and growth rates of *P. canaliculata* upon offering multiple choices of macrophytes with various characteristics would be needed for determining the importance of mixing diets of different plant species and different combinations of plant physical and chemical characteristics on life-history traits and fitness of the apple snails. 

### 4.2. Snail Fecundity Performance

As similar to the growth response, quality of macrophytes play an important role on determining the reproductive output of *P. canaliculata*, *i.e*., number of clutches and clutch size produced per female snail. In this study, plant N, phenolic and cellulose contents were the most influential factors on snail reproductive output. Female snails fed with the most nutritive and least defensive *Ipomoea* exhibited the highest reproductive output. But, females reared on the nutrient-poor and highly-defensive *Acorus* and *Phragmites* had the lowest reproductive output and the clutch size was <30% that of the reported range for *P. canaliculata* from literature (clutch size = 200–300 eggs) [13,18,62]. Our findings were consistent to previous studies on snail fecundity such that egg production of *P. canaliculata* was positively correlated with plant N and P contents, e.g., [16,32]. Other ecological studies also indicated that *P. canaliculata* had lower egg production during food (nutrient)-limited conditions [63,64]. As the fecundity was considered the strongest predictor for invasive success in mollusks [65], *Acorus* and *Phragmites* could provide an inhibitory effect on the clutch size and number of clutch produced by female *P. canaliculata* and hence limit the colonisation extent of this invasive pest in constructed wetlands. 

The offspring quality of *P. canaliculata* was consistent regardless the maternal feeding conditions, except that individuals reared on *Phragmites* produced eggs with significantly lower hatching success (<10%) as compared to individuals fed with the other four macrophytes (~50%). Previous investigations revealed that neither food quality of macrophyte species nor food availability had strong association with egg hatching success [32,62,63,66]. Also, the egg hatching success of *Pomacea* spp. varied greatly in both laboratory and field environments (*P. canaliculata* = 0%–99.0%; *P. maculata* = 0.9%–94.3%). In this study, only 35% of the variation of egg hatching success of *P. canaliculata* was explained by plant phenolic and cellulose contents according to the best subset regression model. This suggested that other important factors, such as temperature, water level, population density and other food conditions could be responsible for controlling the snail egg-hatching success, e.g., [67,68]. 

Accumulated studies supported that the offspring size of *P. canaliculata* was a relatively conservative life-history trait; it was independent of maternal feeding condition and less variable than the offspring quantity [14,63,64,69]. This agreed to our findings that either DM-egg or DM-neonate produced by female snails did not exhibit any difference among the five macrophyte feeding treatments, and all measured plant characteristics did not have any influence on the offspring size. Total clutch weight laid by female *P. canaliculata* per day decreased significantly under food-limiting conditions (high food level: 0.23 ± 0.10 g·d^−1^, low food level: 0.12 ± 0.04 g·d^−1^, *p* < 0.01); but the efficiency of transforming ingested food into eggs remained constant (high food level: 0.022 ± 0.008, low food level: 0.015 ± 0.007, *p* > 0.1) [63]. Thus, food scarcity could affect the snail reproductive output (*i.e*., clutch size); but this did not affect the offspring quality (*i.e*., egg size). Decrease in food availability could induce female *P. canaliculata* to change their strategy by allocating their energy to maintain the egg size and neonate survival time but sacrificing the clutch size [64]. According to our findings, the experimental females were still capable of producing an average of 0.5–1 egg clutch during the first two weeks of the feeding treatments with the nutrient-poor and highly defensive *Phragmites* and *Acorus*. This indicated that female snails could allocate their maternal reserve, resulting from feeding on other palatable food in the field before they were collected for the present experiment, to support their reproduction when facing short-term shortage of palatable food. Such adaptive traits of the apple snails are beneficial for maximising the fecundity and offspring survival, and sustaining the populations in the highly variable wetland environments in particular constructed wetlands with low habitat stability and intensive human disturbance [7,62,70,71].

### 4.3. Implications for Bottom-up Control for the Apple Snails in Constructed Wetlands

As indicated by previous studies that ontogenetic diet shift was not distinctive between juvenile and adult *Pomacea* spp. (e.g., *P. canaliculata* and *P. maculata*), this resulted in strong intra-specific competition among different-sized individuals on food resources [14,57,69,72]. Small-sized *P. canaliculata* had higher foraging capacity than large individuals, and they could therefore cause stronger impacts on macrophytes and greater damage to invaded wetlands [57,73]. However, in constructed wetlands predominantly planted with the macrophyte species of low palatability, e.g., *Phragmites* and *Acorus*, small snails could have higher mortality due to aestivation as compared to adults that exhibited competitive advantages to withstand starvation, see [59,72]. Although our results revealed that the offspring quality of *P. canaliculata* was unaffected by the maternal feeding condition, the reduced growth and offspring quantity of the poorly-fed or aestivated snails in constructed wetlands dominated by the less-palatable macrophytes could result in limitation of invasive success and population growth of *P. canaliculata*.

Many constructed wetlands have been established for wastewater treatment purpose, and the essential roles of macrophytes for pollutant removal in constructed wetlands have been well established [21,74]. However, the massive herbivory of restored macrophytes by the invasive *P. canaliculata* have become a major cause of ecosystem dysfunctioning of constructed wetlands [8,14]. Therefore, planting appropriate macrophyte species in constructed wetlands would be important for not only maximising pollutant removal efficiency, but also maintaining adequate macrophyte species and biomass via bottom-up control of *P. canaliculata*. Comparisons of the pollutant removal efficiency by common macrophytes in constructed wetlands revealed that *Ipomoea* and *Commelina* exhibited only limited capability for uptaking nutrients and heavy metals, but *Phragmites* had markedly high pollutant removal ability and better tolerance to various conditions of constructed wetlands [74,75,76,77]. Although *Ipomoea* has been widely planted in the tropical and subtropical constructed wetlands, it could be inappropriate to plant this macrophyte in constructed wetlands as it represented a highly palatable food source and provided good support for growth and fecundity for the invasive *P. canaliculata*. In contrast, as a native species in Asian wetlands with high pollutant removal ability, *Phragmites* would be a better option for planting in constructed wetlands, in particular designed for wastewater treatment, for enhancing their ecological functions and achieving the bottom-up management of the invasive apple snails [77,78]. It must be noted that extensive planting of *Phragmites* may provide effective control for the invasive apple snails, but *Phragmites* can out-compete other plant species in the constructed wetlands and dominates the floral community resulting in the reduction of habitat heterogeneity and wetland biodiversity. Therefore, this would not be an appropriate strategy for those constructed wetlands created for biodiversity enhancement, habitat mitigation or ecological restoration purposes.

Given the technical difficulty in complete eradication of *P. canaliculata* from invaded constructed wetlands, a well-designed planting program with screening criteria including low N and P contents and high levels of phenolics, cellulose and toughness could be useful for selecting appropriate macrophyte species as bottom-up control agent for the highly competitive *P. canaliculata*. Undoubtedly, extensive planting of any single macrophyte species in constructed wetlands would not be a good management strategy. The present work represented the first step to promote bottom-up control strategy to the invasive *P. canaliculata* in constructed wetlands. We suggest that future research be directed to investigate how different mixtures of multiple macrophyte species affect the life-history traits and population dynamics of *P. canaliculata* so as to develop the multi-species planting guides for effective bottom-up control of the apple snails in the constructed wetlands established for different purposes. 

## 5. Conclusions 

The massive herbivory of restored macrophytes by the invasive *Pomacea canaliculata* have become a major cause of ecosystem dysfunctioning of the constructed wetlands. Our results indicated that *P. canaliculata* exhibited different feeding rates on the five common macrophytes in constructed wetlands and their life-history traits varied greatly upon feeding with different plants. The apple snails reared on *Ipomoea* had the highest feeding and growth rates with highest reproductive output, but all individuals fed with *Acorus* and *Phragmites* showed lowest feeding rates and little growth with poorest reproductive output. The plant N and P contents were important for enhancing palatability and supporting growth and offspring quantity of *P. canaliculata*, whilst toughness, cellulose and phenolic contents had critical deterrent effects on various life-history traits. In the constructed wetlands dominated by the poorly-palatable macrophytes, the reduced growth and offspring quantity of the poorly-fed or aestivated snails could result in limitation of invasive success and population growth of *P. canaliculata*.

Although *Ipomoea* has been widely planted in most constructed wetlands, it could be an inappropriate macrophyte to plant in treatment constructed wetlands as this species not only exhibited limited pollutant uptaking capability but also represented a highly palatable food source for *P. canaliculata*, highly supporting its growth and fecundity. In contrast, *Phragmites* would be a better option for planting in constructed wetlands for enhancing the multiple wetland functions and achieving the bottom-up control of the invasive apple snails due to its high pollutant removal ability, better tolerance to wetland conditions and low palatability to *P. canaliculata*. However, extensive planting of *Phragmites* would result in reduction of habitat heterogeneity and wetland biodiversity of constructed wetlands. Future studies should focus on investigating how different mixtures of multiple macrophyte species affect the life-history traits and population dynamics of *P. canaliculata* so as to develop the multi-species planting guides for effective bottom-up control of the apple snails in the constructed wetlands established for different purposes. 

## Figures and Tables

**Table 1:** Physical and chemical characteristics of the five study macrophyte species.

Macrophyte	Type	N	P	Phenolics	Cellulose	Lignin	Toughness
*Ipomoea*	AC	4.28 ± 0.91 ^A^	0.70 ± 0.17 ^A^	0.22 ± 0.01 ^C^	8.60 ± 1.39 ^C^	1.75 ± 0.90 ^BC^	58.86 ± 16.63 ^D^
*Commelina*	E	3.72 ± 0.19 ^AB^	0.75 ± 0.03 ^A^	1.89 ± 1.26 ^B^	15.34 ± 1.27 ^B^	2.17 ± 0.07 ^B^	79.54 ± 11.35 ^C^
*Nymphoides*	FL	3.23 ± 0.34 ^B^	0.45 ± 0.04 ^B^	2.57 ± 0.05 ^AB^	21.13 ± 1.19 ^AB^	1.13 ± 0.39 ^C^	116.41 ± 20.57 ^B^
*Acorus*	E	2.18± 0.10 ^C^	0.36 ± 0.06 ^B^	3.08 ± 0.07 ^A^	22.11 ± 1.18 ^A^	1.11 ± 0.58 ^C^	176.65 ± 25.44 ^A^
*Phragmites*	E	2.38 ± 0.30 ^C^	0.34 ± 0.05 ^B^	0.30 ± 0.01 ^C^	24.85 ± 1.09 ^A^	6.64 ± 0.57 ^A^	237.69 ± 38.57 ^A^

Unit for N, P, phenolics, cellulose and lignin = percentage with respect to the dry weight of leaf sample; unit for toughness = g. All macrophyte traits were significantly different among the five species (one-way ANOVA; *p* < 0.01). The superscript A, B, C and D represented significant groupings under *post hoc* Tukey comparisons.

**Table 2:** Mean values (±SD) of percentage plant consumption and percentage SH-growth of *Pomacea canaliculata* fed with each of the five macrophyte species during the study period.

Macrophyte	Plant Consumption		SH-Growth	
	Mean % Feeding	*F*	Mean % SH-Growth	*F*
*Ipomoea*	57.18 ± 27.25	147.78 ***	29.82 ± 8.61	78.41 ***
*Commelina*	25.17 ± 8.58	249.76 ***	17.43 ± 3.78	72.56 ***
*Nymphoides*	48.15 ± 18.83	189.65 ***	14.53 ± 5.16	62.23 ***
*Acorus*	2.66 ± 1.07	177.31 ***	1.46 ± 0.92	1.56 ^NS^
*Phragmites*	1.33 ± 0.79	82.49 ***	0.75 ± 0.55	0.55 ^NS^

*** *p* < 0.001; ^NS^ no significance.

**Table 3:** Pearson’s correlation coefficients between the macrophyte traits of the five study species.

Macrophyte Trait	N	P	Phenolics	Cellulose	Lignin
P	0.831				
Phenolics	−0.603	NS			
Cellulose	−0.715	−0.418	0.541		
Lignin	−0.387	−0.513	−0.564	NS	
Toughness	−0.690	−0.705	NS	0.555	0.749

NS = No significance.

**Table 4:** Summary statistics from analysis of best-subset regression comparing each attributes of the life-history traits of *Pomacea canaliculata* to macrophyte characteristics.

Snail Life-History Trait	Constant	Nutrients		Defensive Traits				Adj. *R*^2^
		N	P	Phenolics	Cellulose	Lignin	Toughness	
Feeding rate	0.359		0.364	−0.081	−0.008			0.78
SH-growth	0.080		0.018		−0.002		−0.001	0.84
DM-growth	2.268		0.520		−0.006		−0.012	0.76
No. of clutches per female	4.870	0.127		−0.211	−0.181			0.41
Clutch size	71.429	66.814		−12.914	−1.664			0.60
Egg hatching success	67.449			−11.821	−2.410			0.23

No macrophyte characteristic selected as predictor in the regression model of DM-egg and DM-neonate.
